# The efficacy of tumor debulking surgery is improved by adjuvant immunotherapy using imiquimod and anti-CD40

**DOI:** 10.1186/1471-2407-14-969

**Published:** 2014-12-17

**Authors:** Andrea Khong, Amanda L Cleaver, Muhammad Fahmi Alatas, Ben C Wylie, Theresa Connor, Scott A Fisher, Steve Broomfield, Willem J Lesterhuis, Andrew J Currie, Richard A Lake, Bruce W Robinson

**Affiliations:** School of Medicine and Pharmacology, The University of Western Australia, Perth, Perth, Western Australia; National Centre for Asbestos Related Diseases, Perth, Western Australia; School of Veterinary and Life Sciences, Murdoch University, Perth, Western Australia; Telethon Kids Institute, Perth, Western Australia

**Keywords:** Surgery, Tumor, Debulk, Immunotherapy, Imiquimod, Anti-CD40, Mesothelioma

## Abstract

**Background:**

Tumor debulking surgery followed by adjuvant chemotherapy or radiotherapy is a standard treatment for many solid malignancies. Although this approach can be effective, it often has limited success against recurrent or metastatic cancers and new multimodality approaches are needed. Adjuvant immunotherapy is another potentially effective approach. We therefore tested the efficacy of the TLR7 agonist imiquimod (IMQ) combined with agonistic anti-CD40 in an incomplete debulking model of malignant mesothelioma.

**Methods:**

Established subcutaneous murine ABA-HA mesothelioma tumors in BALB/c mice were surgically debulked by 75% and treated with either: i) saline; ii) intratumoral IMQ; iii) systemic anti-CD40 antibody, or using a combination of IMQ and anti-CD40. Tumour growth and survival were monitored, and the role of anti-tumor CD4 and CD8 T cells in therapeutic responses was determined.

**Results:**

The combination therapy of partial debulking surgery, IMQ and anti-CD40 significantly delayed tumor growth in a CD8 T cell dependent manner, and promoted tumor regression in 25% of animals with establishment of immunological memory. This response was associated with an increase in ICOS+ CD8 T cells and tumor-specific CTL activity in tumor draining lymph nodes along with an increase in ICOS+ CD8 T cells in responding tumours.

**Conclusions:**

We show that the post-surgical environment can be significantly altered by the co-administration of adjuvant IMQ and anti-CD40, resulting in strong, systemic anti-tumor activity. Both adjuvants are available for clinical use/trial, hence this treatment regimen has clear translational potential.

## Background

Surgical removal of solid tumors aims to provide long term, cancer-free survival. Macroscopic tumour eradication is achieved successfully in many cases, however, relapses can and do occur in some patients. This is mainly due to the inability to completely access and resect the primary tumor, or to the existence of micrometastases at the time of surgery. On-going attempts to improve this situation by the use of adjuvant chemotherapy and/or radiotherapy have met with some success, with significant results seen in breast and colorectal cancer, for example [1, 2]. However, in many other cancer types, treatments delivered post surgery have only limited effect and thus new adjuvant approaches are needed [3].

Adjuvant immunotherapy is gaining renewed interest due to the recent success of checkpoint blockade with drugs such as anti-CTLA-4 and anti-PD-1 [4]. Here we examine the benefits, and mode of action, of a combined adjuvant immunotherapy of imiquimod (IMQ) and systemic agonistic anti-CD40 antibody to treat incompletely debulked AB1-HA tumors. IMQ is a potent toll-like receptor-7 (TLR7) stimulator with anti-tumor properties; most importantly, it promotes DC maturation and activation to aid cross-priming of CD8 T cell responses to tumor antigens. To date, IMQ is one of only three TLR agonists that are FDA-approved for use in human cancer [5]. Agonistic anti-CD40 (CP-870,893) further promotes DC-driven cytotoxic T lymphocyte (CTL) responses through its ability to substitute for CD4 T help [6] and has shown some success in clinical trials due to its synergism with chemotherapy [7, 8]. It is also noted for its ability to drive effector cells from the lymph nodes (LN) to the periphery [9], and in combination with IMQ has demonstrated efficacy against mesothelioma in our mouse model [10]. The AB1-HA mesothelioma tumor is one of the few murine tumor models that closely resembles the homologous human disease, in terms of its defined aetiology, biology and clinical behaviour, meaning that the results described in this study are applicable to human tumors [11].

## Methods

### Mice

BALB/c (H-2^d^) (Specific Pathogen Free (SPF), female, 6–8 weeks of age) mice were obtained from the Animal Resources Centre (Western Australia) and maintained under standard conditions at the University of Western Australia (UWA) QEII Medical Centre animal holding facility. All experiments were performed with approval from the UWA Animal Ethics Committee.

### Tumor cells and inoculation

The AB1-HA murine malignant mesothelioma cell line was generated in our lab as described previously [11]. Cells were maintained in RPMI 1640 (Life Technologies, Australia) supplemented with 20 mM HEPES (Sigma-Aldrich, Australia), 0.05 mM 2-ME, 60 μg/ml penicillin (CSL, Australia), 50 μg/ml gentamicin (Pfizer, Australia), 10% foetal calf serum (FCS; Life Technologies, Australia), and 400 μg/ml Geneticin (G418; Life Technologies, Australia). Trypsinised adherent cells were counted and viability assessed by trypan blue exclusion. Cells were resuspended in phosphate buffered saline (PBS) at 5 × 10^6^ cells/ml and 100 μl injected subcutaneously (s.c) into the shaved, right hand flank of mice. In some mice, tumors were inoculated 7 days later to act as size-matched controls *i.e.,* non-debulked tumor size matches debulked tumor size at commencement of treatment. Tumor size was monitored by electronic callipers and calculated by multiplying the length and width to produce tumor area in mm^2^. Mice were euthanised when tumors reached 100 mm^2^ according to UWA Animal Ethics guidelines.

### Surgical debulking

Primary tumors were partially debulked on day 18 post-inoculation when tumors were approximately 50 mm^2^ in size. Mice were anaesthetised by induction under inhalant methoxyflurane (1 ml/20 g) and maintenance under isoflurane with 5% oxygen. The surgical area was sprayed with 70% ethanol and approximately 75% of the tumor was removed, leaving 25% *in situ*. The area was closed using staples (LT-100 liga clips, Ethicon, North Ryde, Australia) or 5/0 vicryl continuous sutures (Ethicon). Mice were placed under a heat lamp for recovery and received 0.5 mg/kg buprenorphine immediately post surgery.

### Treatments

IMQ [Aldara™ (3 M Pharmaceuticals)] was administered by intratumoral (*i.t.*) injection at 50 μg once daily for 6 days starting at the time of surgery. Anti-CD40 (FGK45; Ab Solutions, Perth, Australia) treatment commenced on day 19 at 100 μg administered intraperitoneally (*i.p.*) given every second day for three doses. For cell depletion studies, anti-CD4 (GK1.5) or anti-CD8 (YTS.169) (Ab Solutions, Perth, Australia) was administered from day 17 (1 day pre-surgery), given every second day for a total of three doses. The initial dose was given intravenously (i.v.), followed by two i.p. injections of 150 μg.

### In vivo CTL assay by flow cytometry

*In vivo* tumor-specific CTL activity was measured as previously described [12]. Briefly, spleens and lymph nodes were isolated from BALB/c mice and disaggregated between frosted glass sides, erythrocytes were lysed using PharmLyse (BD) and the remaining lymphocytes were washed well with PBS. Lymphocytes were then divided into two populations, and either pulsed with CL4 peptide (1 μg/ml for 90 mins at 37°C) and labelled with a high dose of carboxyfluorescein succinimidyl ester (CFSE) (5 μM) or un-pulsed and labelled with a low dose of CFSE (0.5 μM). Both cell populations were combined at a 1:1 ratio and adoptively transferred *i.v.* into recipient tumor-bearing animals. Twenty hours after transfer, lymphocytes were recovered from lymph nodes and spleens, as described above, analysed by FACS for fluorescence intensity staining in the FITC channel. The percentage of tumor-specific CTL was calculated by dividing the percentage of un-pulsed cells (CFSE lo) by the percentage of CL4-pulsed target cells (CFSE hi).

### Flow cytometric assessment of T cell activation

For flow cytometric analysis, spleens, lymph nodes and tumors were harvested and processed into single cell suspensions. The axillary and inguinal lymph nodes were pooled for the tumor flank (draining LNs) and healthy contralateral flank (non-draining LNs). Tissues were disaggregated by rubbing between frosted glass slides. Erythrocytes were lysed using Pharmlyse (BD Biosciences, Australia). Cells were filtered by passing through a 70 μm mesh, then surface-stained using the following antibodies; CD4 PE-Cy7 (eBioscience; Cat. 25-0042-82), CD8 PE-Cy5.5 (abcam; Cat. 37928) and ICOS APC (Biolegend; Cat. 313510). Data were acquired on a FACSCantoII (BD Biosciences, Australia) by collecting 100,000 events in the lymphocyte gate, and analysed using FlowJo software (Treestar, USA) for the percentage of CD4^+^ and CD8^+^ T cell subsets within the lymphocyte gate, and the percentage of each subset expressing ICOS.

### Statistical analysis

**Figure 1:**
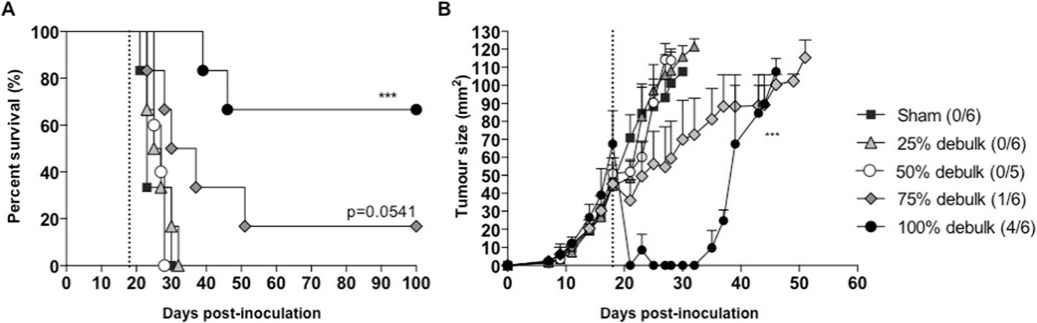
**75% debulk results in delayed residual tumor outgrowth.** BALB/c mice bearing AB1-HA tumors underwent surgical debulking of different percentages on day 18 post-tumour inoculation (dotted line). **A**. Survival and **B**. Residual tumour outgrowth were monitored. Surviving mice shown in brackets. ***p < 0.001 compared to untreated; log-rank test.

**Figure 2:**
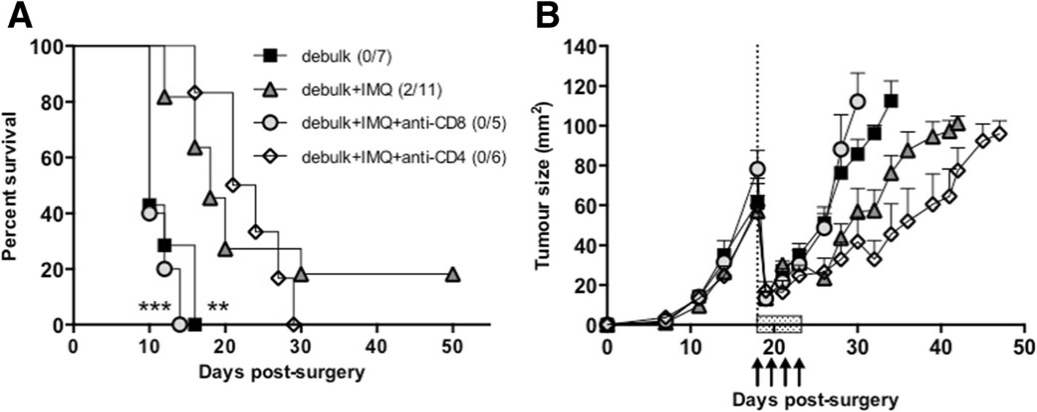
**CD8 T cells are absolutely required for the survival benefit of combined surgery and IMQ treatment.** BALB/c mice bearing AB1-HA tumors were left untreated or partially (75%) surgically debulked (dotted line) of tumor, then treated with immunotherapy (IMQ; 50 μg i.t. q1dx6 on day of surgery; shaded box) and anti-CD4 (GK1.5) or anti-CD8 (YTS.169) specific antibodies (arrows) administered from day 17 (1 day pre-surgery), given every second day for a total of three doses. The initial dose was given intravenously (i.v.), followed by two i.p. injections of 150 μg. **A)** Survival curves of treatment groups as indicated. Number of survivors/total number of animals in parentheses. ***p =0.0004 comparing 75% debulk + IMQ + anti-CD8 to 75% debulk + IMQ, **p = 0.0014 comparing debulk alone to debulk + IMQ; log-rank test **B)** Tumor growth curves of treatment groups as indicated (survivors with complete tumour regression not included).

**Figure 3:**
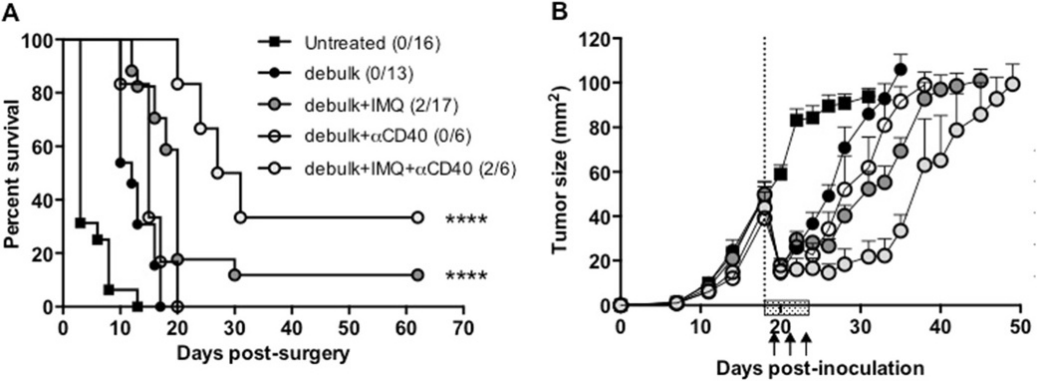
**Anti-CD40 therapy boosts the survival benefit of combined tumor debulking surgery and local IMQ therapy.** BALB/c mice bearing AB1-HA tumors were left untreated or partially (75%) surgically debulked (dotted line) of tumor, then treated with immunotherapy (IMQ; 50 μg i.t., q1dx6; shaded box) and α-CD40 antibody (FGK45; 100 μg i.p. q2dx3, starting one day after surgery and IMQ treatment; arrows). **A**. Survival curves of treatment groups as indicated. Number of survivors/total number of animals in brackets. ****, *p* < 0.0001, comparing (debulk + IMQ) and (debulk + IMQ + α-CD40) to debulk only; log-rank test **B**. Tumor growth curves of treatment groups as indicated.

**Figure 4:**
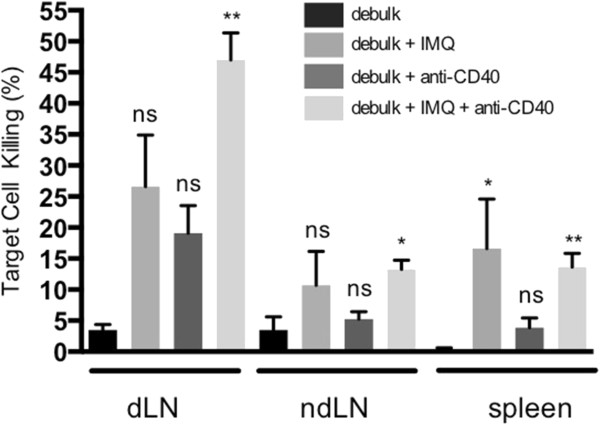
**Anti-CD40 enhances local but not systemic CTL responses induced by IMQ.** BALB/c mice bearing AB1-HA tumors were left untreated or partially (75%) surgically debulked of tumor then treated with immunotherapy (IMQ; 50 μg i.t. q1dx6 on day of surgery and/or anti-CD40; 100 μg i.p. q1dx1, starting one day after surgery). CFSE-labelled, CL4-pulsed and un-pulsed target cells were injected i.v. on day 25 post inoculation and 18 hours later the dLN, ndLN and spleen were harvested and analysed by flow cytometry to assess levels of CTL activity. ns = not significant, *p < 0.05, **p < 0.01 compared to untreated; Kruskal-Wallis test with Dunn’s correction for multiple comparisons.

**Figure 5:**
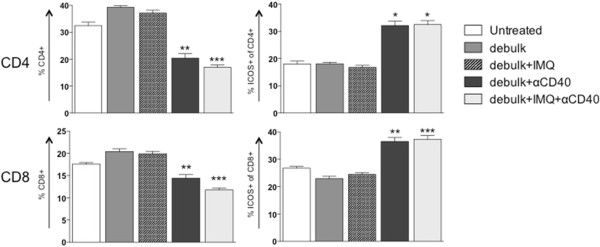
**T cell proportions and activation in the spleen following combined debulking surgery with IMQ and/or anti-CD40.** BALB/c mice bearing AB1-HA tumors underwent debulking surgery and treatment with IMQ (50 μg i.t. q1dx6 on day of surgery) and/or anti-CD40 (100 μg i.p. q1dx1, starting one day after surgery). On day 26, the spleens were removed for analysis of T cell subsets by flow cytometry. CD4+ and CD8+ T cells were identified as% of total lymphocytes (based on forward and side scatter), and analysed for ICOS expression (activation status) as% of total CD4+ or CD8+ T cells. *p < 0.05, **p < 0.01, ***p < 0.001 compared to untreated; Kruskal-Wallis test with Dunn’s correction for multiple comparisons.

**Figure 6:**
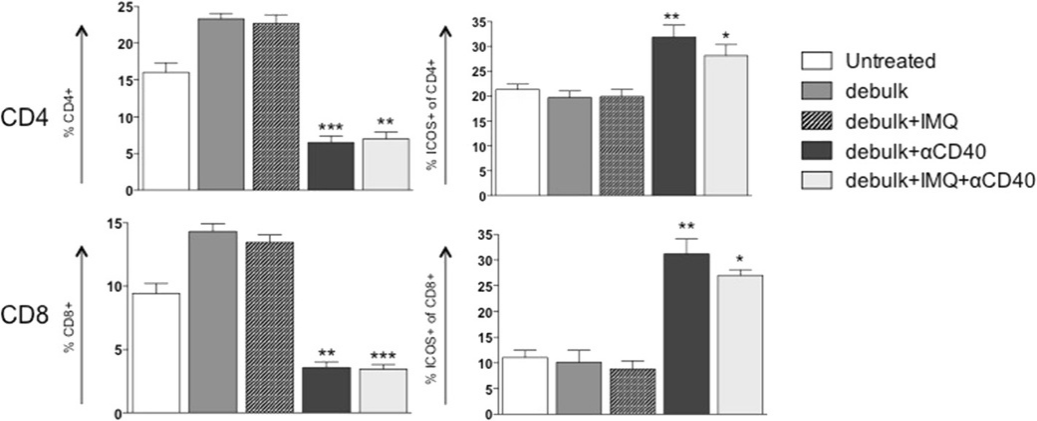
**T cell proportions and activation in the draining lymph nodes following combined debulking surgery with IMQ and/or anti-CD40.** BALB/c mice bearing AB1-HA tumors underwent debulking surgery and treatment with IMQ (50 μg i.t. q1dx6 on day of surgery) and/or anti-CD40 (100 μg i.p. q1dx1, starting one day after surgery). On day 26, the draining lymph nodes were removed for analysis of T cell subsets by flow cytometry. CD4+ and CD8+ T cells were identified as% of total lymphocytes (based on forward and side scatter), and analysed for ICOS expression (activation status) as% of total CD4+ or CD8+ T cells. *p < 0.05, **p < 0.01, ***p < 0.001 compared to untreated; Kruskal-Wallis test with Dunn’s correction for multiple comparisons.

**Figure 7:**
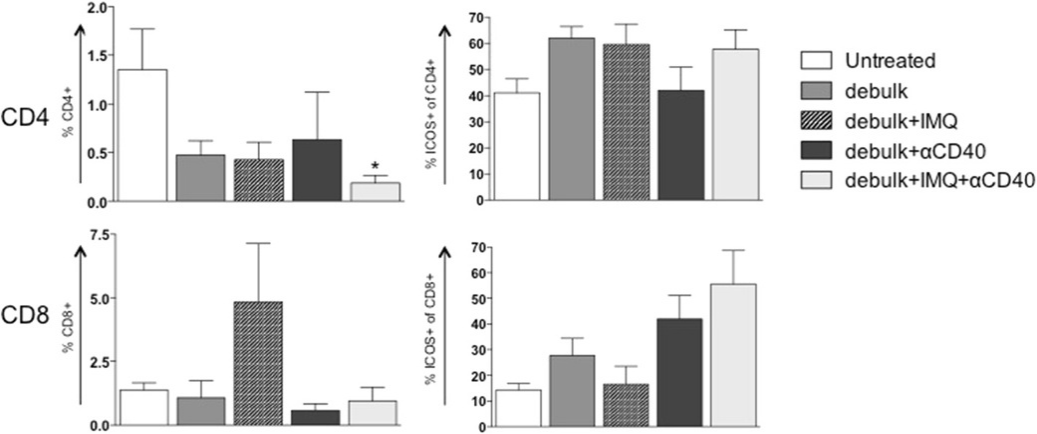
**T cell proportions and activation in the tumor following combined debulking surgery with IMQ and/or anti-CD40.** BALB/c mice bearing AB1-HA tumors underwent debulking surgery and treatment with IMQ (50 μg i.t. q1dx6 on day of surgery) and/or anti-CD40 (100 μg i.p. q1dx1, starting one day after surgery). On day 26, the tumors were removed for analysis of T cell subsets by flow cytometry. CD4+ and CD8+ T cells were identified as% of total lymphocytes (based on forward and side scatter), and analysed for ICOS expression (activation status) as% of total CD4+ or CD8+ T cells. *p < 0.05, **p < 0.01, ***p < 0.001 compared to untreated; Kruskal-Wallis test with Dunn’s correction for multiple comparisons.

Each experiment contained a minimum of 5 mice per group and was repeated at least twice. Statistical analysis was performed using GraphPad Prism software (San Diego, CA, USA). Tumour growth curves were analysed using the Mann–Whitney non-parametric test and the log rank test was used for Kaplan Meier survival plots (Figures 1, 2, 3 & 4). The Kruskall-Wallis test with Dunn’s correction for multiple comparisons was used to compare differences in% CTL or% lymphocytes between treatment groups (Figures 5, 6 & 7). Differences were considered significant if the p value was less than 0.05.

## Results

### Partial debulking of 75% of the tumor mass provides the best scenario for adding adjuvant immunotherapy

We first established our model of partial tumor debulking by investigating the effects of removing different proportions of tumor mass on the growth of residual tumor and overall survival (Figure 1). Complete resection of the tumor was attempted and achieved in 4 out of 6 mice and resulted in tumor-free survival >100 days (Figure 1A). Tumor outgrowth in the remaining 2 mice for which complete debulking was ineffective was delayed for ~15 days before rapid growth of residual tumour cells over a further 15 days (Figure 1B). When 75% of the tumor was debulked, <20% of animals had tumor-free survival (Figure 1A) but tumor outgrowth in the remaining animals was relatively slow, with the last mouse culled 32 days post surgery (Figure 1B). In contrast, when 50% or less of the tumor was debulked, tumor growth rates and tumor-free survival benefit were indistinguishable from sham-treated animals (Figure 1A & B). There were no changes in the proportion of activated (ICOS+) CD4 or CD8 T cells in the different surgical debulking groups either in the tumor itself or in the peripheral lymphoid organs (data not shown). We used a modified dye dilution assay to test the functional efficacy of CTL *in vivo* [13]. In all cases, effector CTL responses were below the limits of detection (data not shown) suggesting that surgical debulking alone does not lead to systemic immune activation even in the context of slower tumor growth rate. Debulking of 75% of tumor mass was chosen for subsequent experiments to determine if a window of opportunity for adjuvant immune therapy exists after incomplete tumor resection.

### Local IMQ therapy boosts the survival benefit of tumor debulking surgery in a CD8 dependent manner

To investigate the efficacy of IMQ in the post-surgical environment, we compared the outgrowth of residual, debulked tumors to size-matched, unresected control tumors (inoculated 7 days later than debulked mice) following administration of IMQ. Intratumoral injections can potentially cause immune and inflammatory stimulation, however we have shown in previous studies that i.t. administration of saline does not affect subcutaneous tumor outgrowth, nor cause the release of type I IFNs or promote antigen-specific immune responses [10]. Control i.t. injections were therefore not included in the present study. There was no significant difference between the treatment groups in terms of the rate of tumor outgrowth, however, debulk plus adjuvant IMQ produced a growth delay of up to 30 days with 20% survival in excess of 50 days (Figure 2). These surviving mice were also shown to have developed tumor-specific memory, as demonstrated by resistance to rechallenge with parental AB1 tumor cells inoculated into the contralateral flank.

Cell depletion studies confirmed an absolute requirement for CD8 T cells in the anti-tumor response elicited by adjuvant IMQ therapy. In contrast to CD4 T cell depletion, which did not result in any long-term survivors, depletion of CD8 T cells resulted in a significantly decreased survival and increased residual tumour outgrowth compared to debulk plus IMQ ([14] and Figure 2).

### Agonistic anti-CD40 therapy boosts the survival benefit of combined tumor debulking surgery and local IMQ therapy

A lack of CD4 T cell infiltrate has previously been reported for locally administered IMQ, and can be improved by co-administration of agonistic anti-CD40 antibody [10]. Thus our next step was to determine whether the addition of anti-CD40, administered on the day of surgery, could further improve the outcome relative to debulking surgery and adjuvant IMQ. Addition of anti-CD40 to debulking surgery led to a survival delay of approximately 7 days more than untreated mice. This was comparable to debulk alone, which conferred a survival delay of approximately 5 days more than untreated mice (Figure 3A). Residual tumor outgrowth following debulk plus anti-CD40 was delayed by 2–3 days compared to debulk alone (Figure 3B). In contrast, the combination of debulk plus IMQ plus anti-CD40 produced a significant survival advantage of 10 days over debulk alone (p < 0.0001; Figure 3A), and residual outgrowth was markedly slower than either debulk plus IMQ, or debulk plus anti-CD40 (Figure 3B). Thus, the combination of anti-CD40 therapy and IMQ provided a significant advantage to debulking surgery compared to surgery alone, and was superior to debulk plus single adjuvant therapy with either IMQ or anti-CD40.

### Anti-CD40 enhances CTL responses induced by IMQ following debulking surgery

We next investigated the effect of adding anti-CD40 to the CTL responses and activation status of specific T cell subsets. The addition of IMQ to debulking surgery led to increased CTL in the dLN, ndLN and spleen, compared to debulk alone (Figure 4). Similarly, addition of anti-CD40 to debulking surgery led to increases in CTL in the dLN, ndLN and spleen (19%, 6% and 4% respectively) compared to debulking alone (3%, 3% and 1%, respectively) (Figure 4). Combination treatment of debulking surgery with IMQ and anti-CD40 resulted in significant increases in CTL killing to over 45% in the dLN (p < 0.001), 15% in the ndLN (p < 0.05) and 15% in the spleen (p < 0.01) (Figure 4). These results demonstrate that the triple therapy produces CTL responses that are greater than debulk alone, or debulk with either IMQ or anti-CD40 therapy.We also analysed T cell subset distribution and activation status in the dLN, spleen and tumour following each of the different treatment combinations. Overall, there was no significant difference between CD4 and CD8 T cell proportions and activation following debulking surgery compared to untreated mice, in all tissues analysed (Figure 6). In the dLN, we found that mice receiving debulk + IMQ exhibited a slight increase in the percentage of both CD4 and CD8 T cells compared to debulk alone, while the activation status (ICOS expression) of CD4 T and CD8 T cells remained unchanged compared to both debulk alone and untreated mice (Figure 6). In contrast, anti-CD40 treatment (either with debulk alone, or debulk + IMQ) resulted in significantly decreased percentages of CD4 and CD8 T cells in the dLN (p < 0.001), while activation status of both subsets was significantly increased, particularly CD8 T cells (p < 0.001) compared to debulking alone (Figure 6). Similar trends were observed in the spleen, with addition of anti-CD40 treatment leading to significantly decreased percentages of CD4 and CD8 T cells and increased ICOS expression in both subsets compared to debulk only (Figure 5).In the tumor, we observed an overall trend towards lower percentages of CD4 T cells and higher activation status in all treatment groups compared to untreated, with the only significant decrease observed in the debulk + IMQ + anti-CD40 group (CD4 T cells, p < 0.05; Figure 7). CD8 T cell proportions in all treated groups were similar to untreated, apart from a 3% increase in the debulk + IMQ treated group. There was also a trend towards increasing CD8 T cell activation, with >10% increase following debulk + anti-CD40 and >20% increase following debulk + IMQ + anti-CD40 (Figure 7).

Taken together, these data indicate that following anti-CD40 administration the proportion of T cells did not expand but were significantly more activated. An important observation was that the dLN and spleens of all mice treated with anti-CD40 were more than doubled in size compared to other treatment groups (not shown). This may account for the lower percentage of immune cells, as a proportion of total splenocytes, found in these mice. Thus anti-CD40 enhances the effect of debulk alone through its ability to activate T cell subsets, and is able to boost the additional action of IMQ by increasing CTL in the dLN.

## Discussion

Occult residual tumor at the resection site or metastatic deposits can often limit the success of surgery as a cancer treatment. Immunotherapy represents a potentially useful adjuvant after cancer surgery to eliminate any remaining tumor cells by inducing antigen-specific anti-tumor activity and stimulating the patient’s immune response to attack residual tumour cells. In this study we investigated agents that target the dendritic cell (DC) because of the powerful role of the DC in stimulating and orchestrating anti-tumor responses. The combined regimen of TLR7 stimulation (IMQ) and activating immunotherapy (anti-CD40) represent two powerful means of activating DCs [10].

### Benefits of administering immunotherapy after surgical debulking

We hypothesised that in cases where complete tumor resection is not possible, there is an optimal amount of tumour to debulk that provides the best environment for adjuvant therapy. As indicated in our model, this is approximately 75% of medium-sized, established tumours. This result is encouraging as it suggests that even with larger-sized, difficult to access, or more advanced tumours where surgery may not be considered the best course of treatment *i.e.,* mesothelioma, if the majority of it can be removed then this will provide a good opportunity for adjuvant immunotherapy to work.

### Surgery provides an opportunity for local therapy

There are several theoretical advantages of providing immunotherapy in a post-surgical setting; the post-surgical environment is altered due to the presence of wound-healing inflammatory mediators, while cytoreduction removes tumor suppressive elements [15] and leads to smaller tumors which are generally more susceptible to immunotherapy [16, 17]. Surgery provides access to the tumor site and thus presents an opportunity for drugs to be administered directly into the tumor, an approach often not possible due to the deep location of many tumors within the body. On a physiological level, localised drug delivery may result in increased potency at the required site of action while at the same time reducing systemic toxicity [18]. The other positive aspects of localised intratumoral drug delivery include the potential conversion of the tumor into its own vaccine [19–21] as well as the potential to produce a systemic effect.

In our model of mouse mesothelioma we have previously tested a variety of dosing regimens and routes and identified that the optimal method of administration of IMQ was to deliver it directly into the tumor on consecutive days [10, 14]. In the current study we found that there was a clear survival advantage with the co-administration of intra-tumorally injected IMQ following debulking surgery, in part due to increased CTL and CD8 T cell activation and the generation of immunological memory. Importantly, given the that surgery can also be potentially immunosuppressive, we note that in fact the act of surgery itself did not adversely affect the efficacy of adjuvant IMQ. This suggests that IMQ and potentially other immune-potentiating agents are suitable drugs for administration post surgery.

### Addition of anti-CD40 improves the anti-tumor response via release of effector T cells

The concept of combining immunisation with co-stimulation has been explored in several murine cancer models. We have previously shown that agonistic anti-CD40 is effective in a number of post-operative settings [22, 23], and that IMQ and anti-CD40 may be combined effectively to treat tumors [10, 22, 24, 25]. In this study, IMQ and anti-CD40 were chosen specifically for their ability to promote tumor-specific CD8 T cell egress from the draining lymph nodes [9] and their likely role in improving CD8 entry/function at the effector site [26]. We found that CTL responses were significantly enhanced in the periphery (ndLN and spleen), and more so in the local dLN following surgery with IMQ and anti-CD40 (Figure 3). This is in contrast with what has been found by others, e.g., co-administration of IMQ and anti-CD40 is ineffective against intradermal B16 melanomas, and required a combination of TLR3, TLR4 and TLR7 stimulation to produce 50% tumor rejection [25]. This highlights the effectiveness of this combination in our model, but in general suggests a multi-targeted approach may be ideal. Indeed, a preclinical surgical study using the renal cell carcinoma model has shown that anti-CD40 may be successfully combined with IL-2 to orchestrate effective DC and CD8 T cell response against distal tumours [27]. It may also indicate a need to overcome residual tumor immune suppression. A recent preclinical study showed that low dose anti-CTLA-4 delivered *i.t.* caused a reduction in tumor-associated Tregs and regression of a distal tumor [18]. For future studies it would be interesting to incorporate a combination of immunotherapies that ‘accelerate’ the immune response (i.e., anti-CD40) and release the ‘brakes’ on existing responses (i.e., anti-CTLA-4, anti-PD-1) to produce an even stronger immune response after surgery [28, 29].

## Conclusion

**Figure 8:**
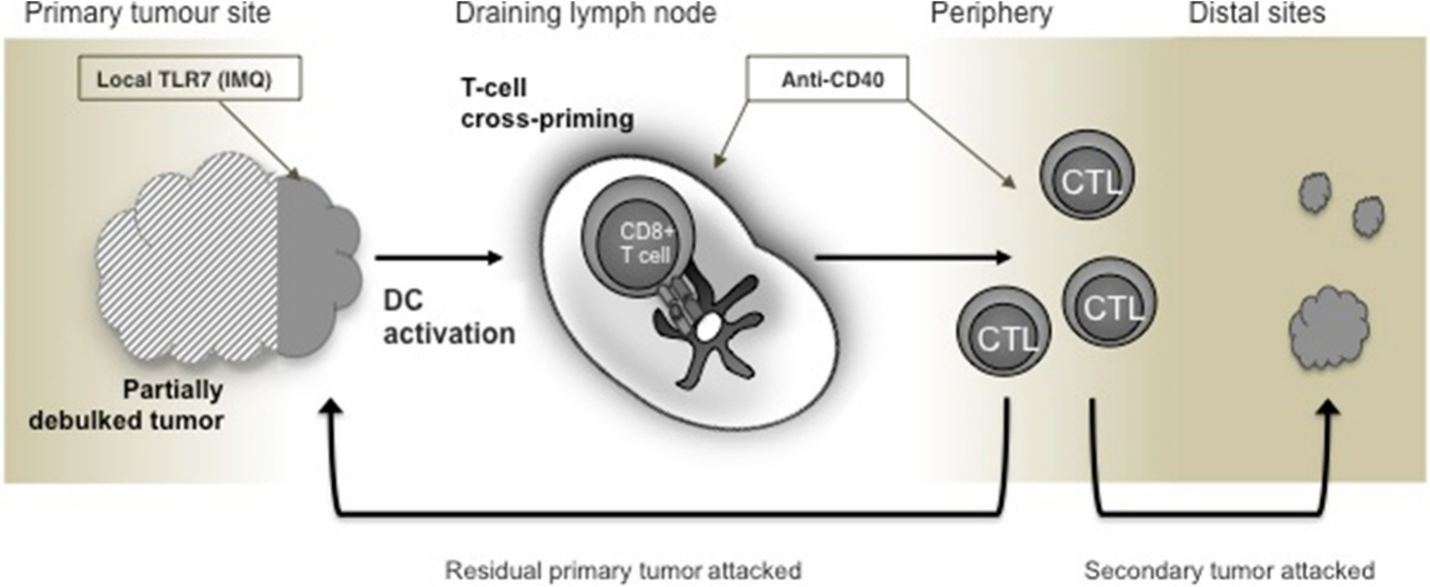
**Immune accelerators acting on remaining tumor after debulking surgery induce a systemic anti-tumor response capable of attacking local residual tumor.** In cases of incomplete debulking surgery, local delivery of IMQ into the tumor site combined with systemic delivery of activating anti-CD40 is an effective approach to promote DC activation and cross-priming of CD8 T cells, leading to a systemic anti-tumor response capable of attacking residual primary tumor deposits and, potentially, secondary metastatic deposits.

We have shown that debulking surgery alone alters the dynamics of the immune response by changing the proportions of CD4 and CD8 T cells. In the context of this new post-surgical environment, cells of the immune system can be further activated by the synergistic combination of TLR7 stimulation and anti-CD40, resulting in improved outcomes. A summary of how these agents are believed to work together is shown in Figure 8. Importantly, these data indicate the unique post-surgical environment should be considered an opportunity to administer immune-modulating agents to target inoperable, residual or metastatic tumor, an approach warranting future clinical study. Further investigations are needed to identify which therapies are beneficial for which cancers, and what are the mechanisms or cell types are involved in the response. Nevertheless, this study indicates the potential for adjuvant therapies to change the course or nature of surgical management for solid cancers.

## Authors’ information

Co-authors: Amanda L Cleaver, The University of Western Australia, Australia, Amanda.cleaver@uwa.edu.au; Muhammad Fahmi, The University of Western Australia, Australia, fahmialatasdr@gmail.com; Ben C Wylie, Telethon Kids Institute, Australia, bwylie@ichr.uwa.edu.au; Theresa Connor, Telethon Kids Institute, Australia, tconnor@ichr.uwa.edu.au; Scott A Fisher, The University of Western Australia, Australia, scott.fisher@uwa.edu.au; Steve Broomfield, Murdoch University, Australia S.Broomfield@murdoch.edu.au; Willem J Lesterhuis, The University of Western Australia, Australia, willem.lesterhuis@uwa.edu.au; Andrew J Currie, Murdoch University, Australia, A.Currie@murdoch.edu.au; Richard A Lake, The University of Western Australia, Australia, Richard.lake@uwa.edu.au; Bruce W Robinson, The University of Western Australia, Australia, Bruce.robinson@uwa.edu.au.

Andrea Khong and Amanda L Cleaver are the equal first authors.
